# O-GlcNAcylation as a Metabolic Integrator in Cardiovascular Physiology and Disease

**DOI:** 10.3390/ijms27114662

**Published:** 2026-05-22

**Authors:** Saima Shakil Malik, Xinchun Pi, Liang Xie

**Affiliations:** Department of Medicine, Cardiovascular Research Institute, Baylor College of Medicine, Houston, TX 77030, USA; saima.shakil@bcm.edu (S.S.M.); xinchun.pi@bcm.edu (X.P.)

**Keywords:** *O*-GlcNAcylation, heart failure, glycosylation, cardiovascular disease

## Abstract

*O*-GlcNAcylation is a ubiquitous post-translational modification regulated by *O*-GlcNAcase (OGA) and *O*-GlcNAc transferase (OGT) in response to environmental and genetic alterations. It occurs in the nucleus, mitochondrion, and cytoplasm and is implicated in cardiovascular disease (CVD) development. *O*-GlcNAcylation modulates diverse cellular processes, including metabolic pathways, signaling networks, and transcriptional programs. Acute increase in *O*-GlcNAcylation serves as an adaptive response that preserves cardiac function, whereas chronic elevation leads to persistent metabolic dysregulation and promotes pathological cardiac remodeling. In this review, we provide a comprehensive overview of the role of *O*-GlcNAcylation across diverse disease contexts. We also summarize the current understanding of its complex interplay with CVD, including the underlying mechanisms. Finally, we highlight existing knowledge gaps and discuss the therapeutic potential of targeting *O*-GlcNAcylation in various cardiovascular events, emphasizing key priorities for future research.

## 1. Introduction (Global Burden of Heart Failure, Introduction to O-GlcNAcylation and Rationale)

Cardiovascular disease (CVD) is the leading cause of morbidity and mortality worldwide. It accounts for approximately 16% of all disease burden and is responsible for 437 million all-ages disability-adjusted life years (DALYs) and 19.2 million deaths in 2023. From 1990 to 2023, CVD-related burden increased by 42.9% in males and 28.6% in females, respectively, with a notable acceleration from 2010 [1]. Although advances in molecular genetics have substantially improved our understanding of CVD pathogenesis, they remain insufficient for accurately predicting and effectively intervening in cardiovascular events. Therefore, there is an urgent need to identify novel etiologic drivers and define the underlying pathological mechanisms to improve risk prediction and prevent future CVD events.

Protein post-translational modifications rapidly transform protein function, interactions, turnover, and localization, thereby contributing to proteomic diversity. According to different reports, approximately 20% to 50% of proteins are glycosylated, making glycosylation one of the most prevalent post-translational modifications, in which carbohydrate moieties are covalently attached to specific amino acid residues [2,3,4]. In mammals, at least nine distinct monosaccharides are involved in the initial attachment to proteins through linkage to nine different amino acids. A wide range of carbohydrate structures, known as glycans, can then be further modified by sulfation, acetylation, and phosphorylation, thereby generating extensive structural diversity [2,5,6]. This organization produces a level of molecular diversity that exceeds what can be achieved through nucleic acids or amino acids alone [7]. In 1976, Langer et al. reported that treatment of cardiomyocytes with neuraminidase, which removes cell-surface sialic acid residues (acidic sugars), led to an increased Ca^2+^ (calcium) exchange rate across the plasma membrane [8]. It was the first report demonstrating that the sugar moieties of cell-surface glycoproteins may regulate ion exchange in cardiomyocytes. Later, they discovered that eliminating sialic acid residues from neonatal cardiomyocytes significantly altered Ca^2+^ permeability without affecting K^+^ (potassium) ion flux [9]. These studies shed light on the importance of cardiomyocyte glycoproteins in normal cardiac function and their involvement in contractile dysfunction under various pathological conditions.

**Figure 1:**
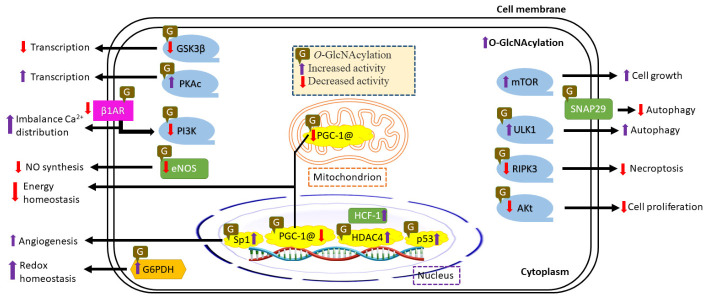
Types, subcellular distribution, and functional roles of O-GlcNAcylated proteins. *O*-GlcNAcylated proteins, primarily localized in the nucleus, cytoplasm, cell membrane, and mitochondria, include transcription factors and coactivators (yellow), protein kinases (blue), membrane receptors (pink), metabolic proteins (orange), and other signaling molecules (green). These proteins perform vital roles in gene transcription, epigenetic regulation, cell cycle control, energy metabolism, vascular function, NO production, Ca^2+^ homeostasis and redox balance. They can be *O*-GlcNAcylated (depicted as brown squares labeled ‘G’), and their functions and activities can be directly altered by this modification. Increased *O*-GlcNAcylation levels modulate SNAP29 and mTOR signaling through distinct mechanisms. Purple arrows represent proteins with increased activity following *O*-GlcNAcylation, whereas red arrows indicate decreased activity after *O*-GlcNAcylation. RIPK3: Receptor-interacting protein kinase 3; GSK-3β: Glycogen synthase kinase-3β; PI3K: Phosphoinositide 3-kinase; PKAc: PKA catalytic subunit; eNOS: Endothelial nitric oxide synthase; β1AR: β1-adrenoceptor, G6PDH: Glucose 6-phosphate dehydrogenase; Sp1: Specificity protein 1; HCF-1: Host cell factor-1; PGC-1α: Peroxisome proliferator-activated receptor-γ coactivator-1α; HDAC4: histone deacetylase 4; SNAP29: Synaptosomal-associated protein 29; mTOR: mammalian target of rapamycin; ULK1: Unc-51-like autophagy activating kinase 1.

Glycomics is an expanding area that has generated transformative concepts and opened new therapeutic opportunities. In 1983, Carmen-Rosa Torres and Gerald Hart identified *O*-linked β-*N*-acetylglucosamine glycosylation—termed *O*-GlcNAcylation (*O*-GlcNAc)—in the mitochondrion, nucleus, and cytoplasm of mouse lymphocytes [10,11,12]. Since then, protein *O*-GlcNAcylation has been widely implicated in the progression of cardiovascular dysfunction. However, the findings remain inconsistent, and the exact role of *O*-GlcNAc (Figure 1) appears to depend heavily on the specific disease context [13]. Protein glycosylation is the covalent attachment of complex glycan chains made up of multiple sugar residues to proteins, classically within the secretory pathway. O-GlcNAcylation, however, is an evident intracellular post-translational modification linking the reversible addition of a single N-acetylglucosamine moiety to serine or threonine residues of mitochondrial, nuclear, and cytoplasmic proteins. This distinction is critical, as O-GlcNAcylation varies profoundly in localization, mechanism, and functional dynamics from classical glycosylation.

In this review, we provide a comprehensive overview of the biology of O-GlcNAcylation and its physiological roles in cardiac homeostasis and acute stress responses. We further discuss its involvement in heart failure pathogenesis, with particular emphasis on hypertrophy, mitochondrial dysfunction, oxidative stress, calcium-handling abnormalities, and fibrosis. In addition, we summarize in vivo models with altered O-GlcNAc signaling and discuss its dual nature, being protective in acute settings but detrimental under chronic conditions. Finally, we address therapeutic potential, emerging technologies, current knowledge gaps, and future directions.

## 2. Biology of O-GlcNAcylation

**Figure 2:**
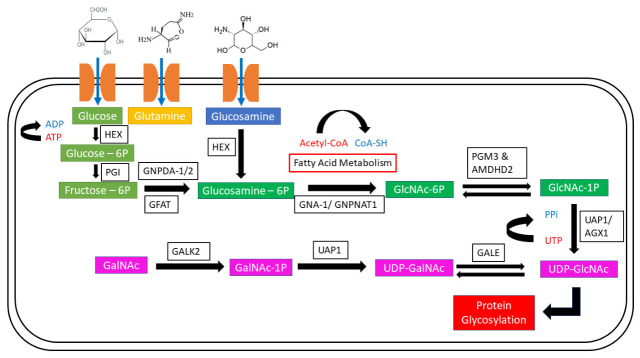
Hexosamine biosynthesis pathway (HBP) and UDP-GlcNAc synthesis. HEX: hexokinase; Glucose–6P: glucose-6-phosphate; PGI: glucose-6-phosphate isomerase; Fructose–6P: fructose-6-phosphate; GFAT: l-Glutamine–d-fructose-6-phosphate aminotransferase; glucosamine–6P: glucosamine-6-phosphate; GlcNAc-6P: *N*-acetylglucosamine-6-phosphate; GNPNAT1: glucosamine 6-phosphate N-acetyltransferase; PGM3: Phosphoacetylglucosamine mutase; UAP1: UDP-*N*-acetylhexosamine pyrophosphorylase; GalNAc: *N*-acetylgalactosamine; UDP-GalNAc: UDP-*N*-acetylgalactosamine; GALE: UDP-glucose-4-epimerase; GNPDA-1/2: Glucosamine-6-phosphate isomerase 1/2; AMDHD2: *N*-acetylglucosamine-6-phosphate deacetylase; PGM3: phosphoglucomutase 3.

The hexosamine biosynthetic pathway (HBP) is a pivotal branch of glucose metabolism that generates uridine diphosphate *N*-acetylglucosamine (UDP-GlcNAc), the essential substrate for protein *O*-GlcNAcylation. In the first step, fructose-6-phosphate is converted to glucosamine-6-phosphate [14] by glutamine–fructose-6-phosphate aminotransferase-1 (GFAT-1). Two genes encode GFAT isoforms, GFAT1 and GFAT2, and a splice variant, GFAT-L, is primarily expressed in skeletal and cardiac muscle [15,16,17]. GFAT activity is regulated by phosphorylation, and both UDP-GlcNAc and glucosamine-6-phosphate act as powerful allosteric inhibitors of GFAT [18]. Spliced X-box-binding protein 1 (XBP1) transcriptionally regulates GFAT in the heart [16]. However, the relative contributions of cardiac GFAT isoforms remain controversial, with some studies identifying GFAT1 as the primary isoform [19] and others suggesting GFAT2 as the predominant regulator of cardiomyocyte O-GlcNAcylation [20]. Through a coordinated series of sequential enzymatic reactions (Figure 2), the HBP results in the synthesis of UDP-GlcNAc.

## 3. Physiologic Roles in Cardiac Homeostasis and Dual Roles of O-GlcNAcylation

Congenital disorders of glycosylation comprise a heterogenous group of rare genetic diseases characterized by defects in O-linked and N-linked glycosylation, resulting in multisystem abnormalities. Approximately 20% of affected patients exhibit cardiac manifestations, including cardiomyopathies, arrhythmias, and structural changes [21], highlighting the significance of protein glycosylation in cardiovascular homeostasis.

Global deletion of glycosyl-transferases or glycoproteins in mouse models often results in embryonic lethality, underscoring their essential roles in cardiac development. Differential expression of glycogenes has been observed between ventricular and atrial cardiomyopathies, and significant differences in N-glycan profiles have been identified between neonatal and adult cardiomyocytes [22]. Age-related decline in cardiovascular function is also associated with alterations in the cardiac glycoproteome, characterized by increased high-mannose N-glycans and reduced complex N-glycans in aged mice [23].

Cardiomyocyte-specific *Ogt* knockout mice exhibit perinatal mortality due to impaired cardiac maturation, whereas adult deletion leads to dilated cardiomyopathy [24]. OGT has also been shown to regulate angiopoietin-1 expression during early cardiac development [25]. In contrast, cardiomyocyte-specific *Oga* deletion does not produce overt cardiac abnormalities within two months, although haploinsufficiency results in age-dependent increases in O-GlcNAc levels [26]. Overexpression of a dominant–negative OGA splice variant in cardiomyocytes increases cardiac O-GlcNAcylation and resulted in hypertrophy, diastolic dysfunction, and adverse remodeling after six months [27]. Conversely, cardiomyocyte-specific OGA overexpression decreases O-GlcNAc levels by ~50% without adverse effects up to three months of age [28].

### 3.1. Acute Cardioprotection of O-GlcNAcylation

Overweight and obese individuals are at high risk of developing cardiovascular diseases [29]. Although the increased prevalence of heart failure (HF) is strongly associated with obesity, several studies have shown that obese HF patients have a better overall prognosis than those with low- or normal BMI, a phenomenon known as “obesity paradox” [30,31,32]. Similarly, acute O-GlcNAcylation plays a protective role during cellular stress. O-GlcNAcylation exerts cardioprotective effects during the acute phase following ischemia-reperfusion (I/R) injury. Elevations of O-GlcNAc levels, achieved by OGT overexpressing or increased HBP flux, enhance cardiomyocyte survival by reducing apoptosis and necrosis after I/R injury [33,34]. Similarly, pharmacologic elevation of O-GlcNAc level using glucosamine or OGA inhibitors improves left ventricular developed pressure (LVDP) and decreases cardiac troponin I release in rodent I/R models [35,36,37,38]. Conversely, OGT or HBP inhibition with alloxan or azaserine eliminates these cardioprotective effects [36,39].

Consistent with these findings, acute increases in cardiac protein O-GlcNAcylation are closely associated with the cardioprotective effects of ischemic preconditioning [40]. Both ischemic preconditioning and remote ischemic preconditioning induce cardio-protection against subsequent prolonged ischemic episodes [41]. These interventions increase cardiac O-GlcNAc levels and reduce myocardial infarct size in both in vivo and ex vivo models [37,42,43,44]. Comparable cardioprotective effects have also been observed in type 2 diabetic animal models under both hyperglycemic and hypoglycemic conditions [45,46,47]. In addition, exposure of isolated human atrial trabeculae to plasma dialysate obtained from healthy individuals subjected to remote ischemic preconditioning improved post-ischemic hemodynamic recovery and was associated with increased protein O-GlcNAcylation levels [42]. Importantly, pharmacologic inhibition of the HBP with azaserine reverses these beneficial effects, supporting the key role for acute O-GlcNAc elevation in mediating cardioprotection during I/R injury.

Mechanistically, transient increases in O-GlcNAc levels exert cardioprotection in part by modulating endoplasmic reticulum (ER) stress responses. The ER plays a central role in protein synthesis, folding, and trafficking [48]. Buildup of unfolded or misfolded proteins induces ER stress and triggers an adaptive mechanism aimed at restoring proteostasis. However, sustained or unresolved stress leads to the activation of pro-apoptotic signaling and cell death [49]. A transient increase in O-GlcNAc attenuates ER stress, as evidenced by reduced activation of ER stress markers such as CCAAT/enhancer-binding-protein [50]. Inhibition or deletion of OGT increases the susceptibility of murine cardiac stem cells to hypoxic injury, whereas enhancement of O-GlcNAc improves cell viability [51].

**Figure 3:**
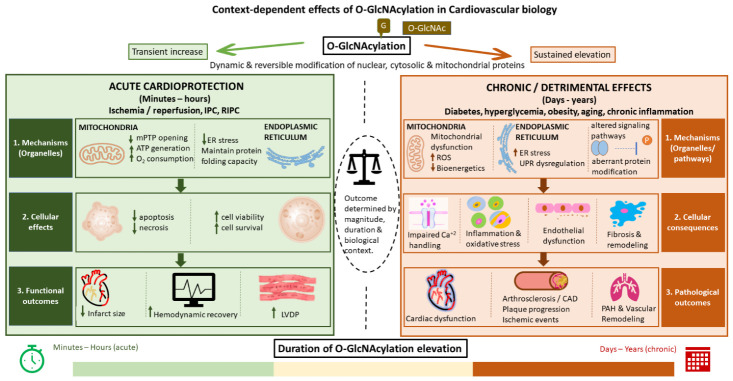
Context-dependent effects of O-GlcNAclation in cardiovascular biology. Schematic showing the dual role of O-GlcNAcylation in cardiovascular physiology and disease. Acute, transient increases (minutes–hours), as seen in ischemia/reperfusion injury and ischemic preconditioning, are cardioprotective, improving mitochondrial function, reducing ER stress, limiting cell death, and enhancing functional recovery. In contrast, chronic elevation (days–years), as observed in diabetes, obesity, aging, and chronic inflammation, promotes maladaptive remodeling, including mitochondrial dysfunction, oxidative stress, impaired calcium handling, endothelial dysfunction, fibrosis, and inflammation, contributing to heart failure, atherosclerosis/coronary artery disease, and pulmonary vascular remodeling. Mechanisms are derived from cellular, animal, and human studies and include both experimentally validated and context-dependent interpretations. The net effect of O-GlcNAcylation depends on its duration, magnitude, and biological context. Upward arrow (↑): increase or activation; downward arrow (↓): decrease or inhibition; IPC: ischemic preconditioning; ER: endoplasmic reticulum; RIPC: remote ischemic preconditioning; UPR: unfolded protein response; mPTP: mitochondrial permeability transition pore; ROS: reactive oxygen species; LVDP: left ventricular developed pressure; ATP: adenosine triphosphate; CAD: coronary artery disease; O_2_: oxygen; Ca^2+^: calcium.

Mitochondrial O-GlcNAcylation also contributes to cardioprotection [13]. OGA inhibition with thiamet G enhances ATP generation and mitochondrial oxygen consumption rate. Moreover, mitochondria isolated from thiamet G-treated rats display increased calcium uptake capacity. This increased calcium uptake capacity reflects a higher calcium threshold for mitochondria permeability transition pore (mPTP) opening following OGA inhibition, supporting a protective role against cell death, as mPTP opening triggers apoptosis [52]. Figure 3 presents acute and chronic effects of O-GlcNAcylation in cardiovascular biology side-by-side, further organized into underlying mechanisms (organelles/pathways), cellular effects, and functional or pathological outcomes.

### 3.2. Chronic O-GlcNAc-Mediated Detrimental Effects

In contrast to the acute protective effects, chronic activation of the HBP and sustained elevation of O-GlcNAcylation contribute to adverse cardiac remodeling [53]. Twenty-two-week-old hyperglycemic ZDF rats display elevated O-GlcNAc and UDP-GlcNAc levels, which are accompanied by impaired cardiomyocyte contraction and relaxation due to altered calcium handling [54]. In contrast, six-week-old rats demonstrate hyperinsulinemia in the absence of overt hyperglycemia and maintain preserved cardiomyocyte mechanical function, suggesting that early metabolic dysregulation precedes functional deterioration.

Left ventricular biopsies acquired from diabetic patients demonstrated substantially increased OGT, OGA, and O-GlcNAc levels compared with non-diabetic myocardium. In addition, total O-GlcNAc levels correlate positively with blood glucose and HbA1c, while inversely correlating with left ventricular ejection fraction (EF) [55]. These results indicate that, during hyperglycemia, nutrient overload increases O-GlcNAc and UDP-GlcNAc levels and is linked with decreased cardiac function. Diabetic mice with overexpressed OGA have improved LVDP and peak rate of relaxation. This confirms that chronic increase of protein O-GlcNAcylation is detrimental in the diabetic heart [56].

Chronic increase of O-GlcNAcylation plays an important role in cardiac hypertrophy (Figure 3), heart failure, and hypertension [57,58,59]. Animal studies with models of aortic banding, myocardial infarction, and hypertension consistently demonstrate increased cardiac protein O-GlcNAcylation [60]. OGA inhibition in muscle strips isolated from post-myocardial infarction failing rat hearts reduces left ventricular contractility, further supporting a maladaptive sustained O-GlcNac signaling. Both rat and mice neonatal cardiomyocytes with increased O-GlcNAc signaling develop features of left ventricular hypertrophy [61]. Moreover, myocardial samples from patients with severe aortic stenosis exhibit 65% higher O-GlcNAc levels than non-ischemic areas from coronary artery disease patients [60]. Hence, these findings indicate that chronic elevation of O-GlcNAcylation contributes to cardiac dysfunction and pathological remodeling in both animals and humans.

Notably, p53, a transcription factor that regulates genes involved in DNA repair and apoptosis, has also been implicated in the inhibition of angiogenesis through multiple pathways. O-GlcNAcylation stabilizes p53, thereby suppressing angiogenesis following cardiac injury [62,63]. Hyperglycemia-induced O-GlcNAcylation of p53 promotes ventricular myocyte apoptosis. Because p53 stability is normally regulated by phosphorylation, O-GlcNAcylation can interfere with this modification and alter p53 turnover and activity [64]. Increased O-GlcNAcylation accompanied by decreased phosphorylation of p38 MAPK (mitogen-activated protein kinase) leads to impaired left ventricular function with increased apoptotic signaling [65,66,67]. Given the critical role of p38 MAPK in p53 activation, O-GlcNAc-mediated modulation of this pathway may contribute to apoptosis-associated cardiac dysfunction.

## 4. GlcNAcylation in Cardiovascular Disease Pathogenesis

The cardiovascular system is highly complex, and O-GlcNAcylation represents critical post-translational modifications that regulate multiple signaling and metabolic pathways. Dysregulated O-GlcNAcylation contributes to metabolic disturbances that impair cardiovascular function, including heart failure [68], cardiac remodeling [59], ischemic heart disease [69], diabetic cardiomyopathy [70], arrhythmia [71], and hypertension [72].

### 4.1. Heart Failure and Cardiac Hypertrophy

Multiple cardiac pathologies ultimately progress to heart failure (HF). Restorative cardiac hypertrophy initially serves as an adaptive response to stress but may transition to HF under sustained pressure overload, and *O*-GlcNAcylation has been implicated in both processes [28,60]. Troponin T, troponin I, and c-Myc exhibit increased *O*-GlcNAcylation in cardiac hypertrophy, leading to maladaptive remodeling [73,74]. Increased *O*-GlcNAcylation has also been detected in left ventricular tissue from HF patients, accompanied by increased protein and expression of HBP-linked enzymes under pressure load conditions [60]. Although substantial evidence connects higher *O*-GlcNAcylation levels with cardiac hypertrophy and HF, the precise molecular mechanisms remain incompletely defined.

Recent studies have explored the role of *O*-GlcNAcylation in cardiomyocyte growth, mitochondrial metabolism, gene expression, and autophagy during HF progression. For example, PGC-1α, a transcription coactivator that regulates mitochondrial biosynthesis and energy metabolism, is functionally modulated by *O*-GlcNAcylation [75]. Decreased PGC-1α activity detected in hypertrophy [76] results from *O*-GlcNAc-mediated inhibition of its expression, thereby impairing fatty acid oxidation and mitochondrial gene expression and triggering metabolic dysregulation (Figure 1) [77]. Increased protein O-GlcNAcylation in OGT transgenic mice is associated with heart failure and dilated cardiomyopathy, where OGT overexpression encourages ventricular arrhythmias and premature death independent of pathological stress, while OGA overexpression eases these lethal effects and preserves myocardial function. These findings suggest that excessive O-GlcNAcylation promotes adverse myocardial remodeling and cardiac dysfunction, potentially through impaired mitochondrial metabolism, including reduced mitochondrial gene activity and decreased complex I expression.

Additionally, the heart exhibits a common feature of compromised cell cycling as a response to heart failure, which has been found to be associated with protein *O*-GlcNAcylation. Higher HBP flux and *O*-GlcNAcylation levels in the cardiomyocytes promote cardiac remodeling and cardiomyocyte growth during cardiac hypertrophy and heart failure through the activation of mTOR, a mechanistic target of rapamycin (Figure 1) [78]. Indeed, mTOR is a key regulator of protein synthesis and cardiomyocyte growth [79] and has been reported to promote cardiac hypertrophic growth [80]. Moreover, cardiomyocyte autophagy is responsible for challenging multiple stimuli that can cause stress in cardiovascular etiology. Interestingly, researchers have illustrated the essential role of protein *O*-GlcNAcylation in cardiomyocytes is to initiate autophagy, entailing the defending role of *O*-GlcNAcylation in HF; this may open new opportunities for HF treatment. This is achieved with the help of an important autophagy initiation regulator known as unc-51, like autophagy-activating kinase 1 (ULK1). ULK1 is *O*-GlcNAcylated, which enhances its activity and promotes the initiation of autophagy in cardiomyocytes (Figure 1) [81].

*O*-GlcNAcylation is also crucial for transcriptional programs involved in inflammatory cytokine and hypertrophic gene expression during HF progression. Increase in protein *O*-GlcNAcylation level in primary cultured mouse cardiomyocytes induces hypertrophic changes and activates PKA (protein kinase A) signaling, which in turn results in a thickened left ventricular wall and cardiac hypertrophy. Specifically, *O*-GlcNAcylation occurs on the catalytic subunit of PKA, increasing its kinase activity (Figure 1). This modification increases the expression of downstream hypertrophic markers such as atrial natriuretic peptide and β-myosin heavy chain [82,83]. These findings underscore the importance of maintaining *O*-GlcNAc homeostasis to prevent maladaptive remodeling in HF.

### 4.2. Ischemia-Reperfusion Injury

I/R injury predisposes the heart to arrhythmia, myocardial infarction, cardiac hypertrophy, and heart failure [84,85,86,87]. *O*-GlcNAcylation modulates cellular survival, redox balance, and therapeutic responsiveness during I/R injury. Researchers have shown that hypoxic acclimatization-induced *O*-GlcNAcylation significantly exacerbates I/R injury through G6PDH (glucose 6-phosphate-de-hydrogenase) activation (Figure 1). It also enhances redox homeostasis, which is recognized as the fundamental role of *O*-GlcNAcylation in hypoxic acclimatization-initiated cardioprotective and antioxidative effects [88]. Additionally, RIPK1/RIPK3/MLKL (receptor-interacting protein kinase 1/receptor-interacting protein kinase 3/mixed lineage kinase domain like) complex plays crucial roles in necroptosis [89]. Sevoflurane-induced *O*-GlcNAcylation of RIPK3 disrupts its interaction with MLKL, restricting necroptosis and attenuating myocardial I/R injury [90].

Glucose–insulin–potassium (GIK) therapy is commonly used in I/R injury. *O*-GlcNAcylation may partially mediate the cardioprotective effects of GIK therapy [91], as insulin increases Akt phosphorylation and triggers Akt-dependent pro-survival signaling [92]. However, in obese individuals, increase in *O*-GlcNAcylation leads to inhibition of insulin-induced Akt phosphorylation and impaired Akt activation, which attenuates insulin-mediated cardioprotection against I/R injury and reduces cell survival, which may explain the decreased efficacy of GIK therapy in obese individuals (Figure 1) [71].

Collectively, current evidence supports a context-dependent role for O-GlcNAcylation, in which moderate increases of O-GlcNAcylation provide protection against I/R-induced cardiac injury, whereas excessive or chronic elevation may attenuate therapeutic benefits.

### 4.3. Diabetic Cardiomyopathy

Diabetic cardiomyopathy is characterized by structural and functional cardiac abnormalities associated with chronic hyperglycemia, including myocardial fibrosis, cardiomyocyte hypertrophy, oxidative stress, left ventricular remodeling, and diastolic dysfunction. Clinically, diabetic cardiomyopathy is also associated with an increased risk of arrhythmias, coronary artery disease, and heart failure [93]. Experimental studies have further implicated excessive protein O-GlcNAcylation in mechanisms linked to diabetic cardiovascular injury, including coronary microvascular dysfunction and cardiomyocyte apoptosis. In particular, studies in type 2 diabetic models demonstrated that elevated O-GlcNAcylation and p53 overexpression were associated with coronary microvascular endothelial dysfunction and impaired cardiac perfusion [94]. In coronary endothelial cells, *O*-GlcNAcylation of p53 inhibits Thr-155 phosphorylation, thereby preventing p53 ubiquitination and degradation [95]. The increase in p53 level promotes cardiac contractile failure, coronary microvascular injury, and cardiomyocyte dysfunction, which are improved by reducing p53 O-GlcNAcylation [94,96,97,98]. It has also been shown that protein *O*-GlcNAcylation disrupts autophagic flux and impairs myocardial function in diabetes. Synaptosomal-associated protein 29 (SNAP29), syntaxin-17 (STX17), and vesicle-associated membrane protein 8 (VAMP8) form a complex reaction during autophagy and are responsible for membrane fusion between lysosome and autophagosome [99]. *O*-GlcNAcylation of SNAP29 inhibits this tri-complex formation and suppresses autophagic degradation, thereby impairing cardiac diastolic function (Figure 1). Modulating SNAP29 O-GlcNAcylation levels may provide a promising therapeutic approach for improving cardiac function [100,101].

Additionally, protein *O*-GlcNAcylation alters intracellular Ca^2+^ handling and accelerates hyperglycemia-induced HF and diabetic cardiomyopathy. β_1_AR is major regulator of the cAMP-PKA-PLB signaling pathway [102]. O-GlcNAc modification of the β_1_AR may decrease its activity and suppress the cAMP-PKA-PLB signaling pathway in diabetic cardiomyopathy. This suppression leads to impaired SERCA2a function, elevated Ca^2+^ influx, and dysregulated Ca^2+^ handling, ultimately promoting cardiomyocyte death. OGT triggers left ventricular diastolic dysfunction and adverse cardiac remodeling in non-diabetic mice; OGA attenuated these effects through modulation of the Ca^2+^-dependent PI3K/Akt/SERCA2a signaling pathway. OGT and OGA also contribute to the regulation of cardiac Ca^2+^ homeostasis under diabetic conditions. OGT-facilitated O-GlcNAcylation impairs PI3K-Akt-SERCA2a signaling and interrupts Ca^2+^ homeostasis, leading to accelerated HF under diabetic conditions. Mechanistically, it leads to decreased PI3K activity and further activation of PTEN, a PI3K inactivator, thereby exacerbating Ca^2+^ dysregulation [55].

Conversely, emerging evidence suggests that protein O-GlcNAcylation may also exert context-dependent cardioprotective effects in diabetic hearts through modulation of epigenetic and stress-response pathways. HDAC4, an epigenetic regulator involved in cardiac remodeling and hypertrophic signaling, undergoes O-GlcNAcylation-dependent cleavage to generate the N-terminal fragment HDAC4-NT, which has been associated with protection against heart failure phenotypes in experimental diabetic models (Figure 1) [103,104]. These findings collectively suggest that maintenance of O-GlcNAcylation homeostasis, rather than uniformly increased or decreased O-GlcNAcylation, may be important in the regulation of diabetes-associated cardiac signaling and remodeling.

### 4.4. Pulmonary Artery Hypertension (PAH)

Emerging evidence suggests that increased O-GlcNAcylation may contribute to pulmonary arterial hypertension (PAH) pathogenesis through effects on vascular cell proliferation, angiogenic signaling, metabolic remodeling, and right ventricular dysfunction. In particular, studies in human idiopathic pulmonary arterial hypertension (IPAH) pulmonary vascular cells have demonstrated a shift toward enhanced glucose utilization and glycolytic metabolism, which may increase flux through the HBP and elevate protein O-GlcNAcylation. Increased O-GlcNAc transferase activity has also been associated with proliferative and angiogenic phenotypes in IPAH endothelial cells, although much of the mechanistic evidence remains cellular and vascular in nature rather than directly demonstrating causality in vivo. [105,106].

Studies in human IPAH pulmonary artery smooth muscle cells (PASMCs) have shown that increased OGT expression and O-GlcNAcylation are associated with enhanced cellular proliferation, partly through mechanisms involving host cell factor-1 (HCF-1) signaling [107]. HCF-1 is a known regulator of cell-cycle progression and mitotic control, although its specific contribution to PAH progression has primarily been examined in cellular models rather than established in vivo [108]. In parallel, vascular endothelial studies in IPAH have linked elevated O-GlcNAcylation with angiogenic phenotypes through specificity protein-1 (Sp1)-dependent VEGF expression and neovascular signaling [106].

Right ventricular (RV) dysfunction represents a progressive maladaptive phenotype and is a major determinant of mortality in PAH. Experimental and clinical evidence suggests that metabolic remodeling is a key contributor to RV failure. Increased protein O-GlcNAcylation has been associated with altered cellular energy handling and mitochondrial stress in cardiac tissue, particularly under conditions of metabolic strain. In models of heart failure, enhanced O-GlcNAc signaling has been linked to impaired mitochondrial homeostasis and glycative stress responses [109]. In PAH-specific experimental models, elevated O-GlcNAcylation has been associated with metabolic derangements and RV dysfunction, including reduced mitochondrial efficiency and contractile performance. Conversely, modulation or reduction of excessive O-GlcNAcylation has been reported to improve mitochondrial function and preserve right ventricular contractility in experimental PAH settings [110].

In addition, studies show that WNK1 (no lysine kinase 1)/Akt activation in PAH induces *O*-GlcNAcylation, which contributes to mitochondrial metabolic dysfunction. Likewise, WNK1 inhibition ameliorates elevated PAH and right ventricular contractile dysfunction, mitochondrial dysfunction, diastolic dysfunction, along with decreasing intracellular *O*-GlcNAcylation levels [111].

It is also noteworthy that decreased NO, a critical pulmonary vasodilator, partially contributes to the increased pulmonary vascular resistance observed in PAH patients [112]. The literature reports an association between eNOS (endothelial nitric oxide synthase) and increased *O*-GlcNAcylation, which affects NO synthesis [113]. Increased *O*-GlcNAcylation at eNOS Ser-615 in PAH inhibits phosphorylation at eNOS Ser-1177, which in turn suppresses eNOS activity. Moreover, *O*-GlcNAcylation of eNOS hinders its dimerization, reduces its stability, and ultimately decreases NO production [114]. Elucidating novel *O*-GlcNAcylation sites and the associated signaling pathways in PAH may provide a foundation for the development of new therapeutic approaches.

### 4.5. Atherosclerosis and Coronary Heart Disease (CHD)

Atherosclerosis is a central pathological process underlying CHD and is a major contributor to global morbidity and mortality, including myocardial infarction, stroke, and peripheral artery disease [115]. CHD development is multifactorial and involves a combination of lipid abnormalities (particularly apolipoprotein B-containing lipoproteins), metabolic disturbances, endothelial dysfunction, and environmental and lifestyle factors such as hypertension, diabetes, and smoking. In patients with diabetes, accelerated atherosclerosis is a key determinant of adverse cardiovascular outcomes, and hyperglycemia is recognized as an important risk factor that contributes to disease progression through multiple metabolic and vascular mechanisms [116]. Hyperglycemia has been shown to regulate vascular A20 expression in diabetic ApoE-null mice via O-GlcNAcylation-dependent ubiquitination and proteasomal degradation [117]. This *O*-GlcNAcylation plays a protective role against inflammation-induced vascular injury by negatively regulating NF-κB signaling cascades [118]. Elevated protein *O*-GlcNAcylation has been shown to inhibit iNOS expression, thereby preventing TNF-α-caused vascular dysfunction [119]. Diabetes and hyperglycemia increase eNOS *O*-GlcNAcylation, which prevents its phosphorylation at activating sites and reduces NO production, therefore contributing to endothelial dysfunction in CVDs [120].

## 5. In Vivo Models of Glycosylation-Related Pathways in Cardiovascular Phenotypes

Over the past years, researchers have revealed various alterations in the HBP flux and protein *O*-GlcNAcylation across cardiovascular diseases, emphasizing the fundamental role of protein *O*-GlcNAcylation in cardiovascular pathophysiology. Notably, *O*-GlcNAcylated proteins, including receptors, transcription factors, protein kinases, and other functional proteins, participate in diverse cellular processes such as signal transduction, energy metabolism, gene transcription, and cell cycle regulation. These modifications influence cardiovascular function through multiple mechanisms, including metabolic reprogramming, electrophysiological signaling cascades, cell cycle control, vascular dysfunction, and stress responses. Table 1 shows that while various in vivo models involve glycosylation-related pathways in cardiovascular phenotypes, direct evidence linking these effects to O-GlcNAcylation remains limited. Most models instead reflect indirect impacts through broader glycosylation networks, metabolic regulation, or extracellular matrix composition. These observations underscore the need for more targeted studies to delineate the specific contribution of O-GlcNAcylation within these complex pathways.

## 6. Emerging Technologies for Studying O-GlcNAc

O-GlcNAcylation exerts context-dependent effects on cardiovascular signaling pathways, with acute elevations promoting adaptive and cardioprotective responses, while chronic elevation leads to maladaptive signaling, metabolic dysregulation, and inflammation. Complementary experimental tools enable temporal dissection of O-GlcNAc dynamics. Low and high O-GlcNAc states exert distinct effects on pathway activity, while various genetic, chemical, and optical tools enable interrogation of O-GlcNAc signaling in cells, as shown in Table 2. The identification and functional characterization of protein O-GlcNAcylation have been driven by advances in analytical technologies and chemical biology, specifically mass spectrometry, RNA-based targeting tools, and metabolic labeling strategies [156].

Mass spectrometry-based proteomics has played a key role in mapping O-GlcNAc modifications at site-specific resolution [157]. O-GlcNAc, being labile in nature, originally limited its discovery via conventional fragmentation methods. However, electron-transfer dissociation tandem mass spectrometry facilitated the preservation of glycosidic bonds during peptide fragmentation [158]. For example, ETD-MS/MS analysis has identified four O-GlcNAcylation sites on β-catenin (S23, T40, T41, and T112). These site-specific modifications provide mechanistic insight into how O-GlcNAc regulates β-catenin function in a context-dependent manner [158]. S23 and T41 are located within regions overlapping phosphorylation sites required for ubiquitination, where O-GlcNAcylation can interfere with the destruction complex and thereby inhibit β-catenin degradation [159]. In contrast, the T112 site lies within the α-catenin interaction domain, implicating O-GlcNAcylation in protein–protein interactions linking cadherins to the cytoskeleton [160].

Functionally, O-GlcNAcylation at S23 has been shown to regulate β-catenin subcellular localization by promoting its redistribution from the nucleus to the plasma membrane through enhanced interaction with E-cadherin [161]. Modification at T41 can prevent phosphorylation at S33/37, thereby inhibiting β-TrCP binding and blocking ubiquitin-mediated degradation. However, O-GlcNAcylation at T40 and T112 has not yet been clearly linked to effects on β-catenin stability or localization. Collectively, these findings highlight a paradoxical role of O-GlcNAcylation in stabilizing β-catenin while simultaneously restricting its nuclear signaling activity [158].

Metabolic chemical reporters, like proteomics advances, helped to visualize and quantify O-GlcNAcylation in living cells [162]. For metabolic chemical reporting, cells are presented with bio-orthogonal sugar analogs; for example, peracetylated N-azido-acetyl-galactosamine is metabolically converted into UDP-GlcNAc analogs, and OGT incorporates them into proteins. The azide-functionalized glycans can then be selectively labeled using click chemistry with alkyne bearing probes, assisting downstream recognition by fluorescence or enrichment for proteomic analysis. These methods allow real-time examining of protein-specific O-GlcNAcylation dynamics in live cells with high temporal and spatial resolution mutually with fluorescence resonance energy transfer (FRET)-based systems [159,163,164]. These advancements facilitate the evaluation of OGT and OGA inhibitors and their effects on global and substrate-specific O-GlcNAc levels.

Recently, RNA-based aptamer technologies have emerged as a robust tool to induce and explore protein-specific O-GlcNAcylation. Dual-specificity RNA aptamers are engineered in a way to concurrently bind OGT and a target protein to promote proximity-driven glycosylation without necessitating global enzyme inhibition [165]. These aptamers are produced via systematic evolution of ligands by exponential enrichment (SELEX) and are capable of selectively enhancing O-GlcNAc modification on specific substrates. Application of dual-specificity aptamers has revealed that targeted O-GlcNAcylation can disclose formerly unknown modification sites and modulate protein interactions in a site-dependent manner. This method provides a highly selective program to examine the functional consequences of O-GlcNAcylation at the level of individual proteins [165].

Together, corresponding technologies such as advanced mass spectrometry, metabolic labeling with bio-orthogonal reporters, and RNA-guided targeting approaches have suggestively extended our ability to detect, quantify, and functionally evaluate O-GlcNAc alterations with greater precision. Recommendations to advance O-GIcNAc biology are provided in Table 3.

## 7. Therapeutic Potential

*O*-GlcNAcylation demonstrates a context-dependent role, acting as either destructive or protective in CVD development, as discussed earlier. However, its therapeutic potential remains largely unexplored in clinical settings. Currently, no drugs specifically targeting *O*-GlcNAcylation have been approved for CVD treatment. However, multiple experimental agents have been used in preclinical studies to alter intracellular *O*-GlcNAcylation levels by inhibiting or increasing the activity of a key enzyme (OGA, OGT, GFAT) or by modulating nutrient flux, suggesting potential therapeutic applications. Commonly used agents in experimental settings include GFAT inhibitors (e.g., *O*-diazoacetyl-*L*-serine [azaserine]), OGT inhibitors (e.g., alloxan), OGA inhibitors (e.g., PUGNAc [*O*-(2-acetamido-2-deoxy-*D*-glucopyranosyliden) amino-*N*-phenylcarbamate]), as well as glucosamine and glutamine [13,166]. Although these compounds have been explored extensively in laboratory studies, they have not progressed to clinical use. Therefore, their safety and efficacy in CVD still require further investigation.

Protein O-GlcNAcylation has also been associated with both physiological and pathological responses following surgical procedures. It may contribute to coronary artery bypass graft failure in CHD patients with T2DM. Moreover, increased *O*-GlcNAcylation in T2DM alters the function of saphenous VSMCs and ECs, leading to maladaptive saphenous vein remodeling and subsequent graft failure [167]. These findings suggest that targeting protein *O*-GlcNAcylation may hold therapeutic potential and warrant further investigation in this context.

## 8. Conclusions

*O*-GlcNAcylation is a ubiquitous post-translational modification that plays a crucial role in modulating cardiovascular function and disease processes. *O*-GlcNAcylation exhibits a dual role, providing protective effects in acute settings but becoming detrimental under chronic conditions. Furthermore, protein *O*-GlcNAcylation has the potential to disrupt energy metabolism, electrophysiological signaling cascades, cell cycle regulation, vascular function, and stress responses by directly modifying proteins or regulating gene expression at the transcription level. Researchers are increasingly using emerging technologies to explore the physiological and molecular alterations induced by *O*-GlcNAcylation in the cardiovascular system. Therefore, improved identification and mechanistic understanding of *O*-GlcNAcylation across cardiac signaling pathways will provide novel insights into its role in CVDs and may facilitate the development of targeted therapeutic strategies.

## Figures and Tables

**Table 1:** Glycosylation- and Matrix-Associated Regulators of Cardiovascular Phenotypes in In Vivo Models.

Functional Class	Protein (Gene)	Model/ System	Cardiovascular Phenotype	Key Process Affected	Ref.
Glycosylation Enzymes and Regulators	Galnt1	KO	Cardiac hypertrophy, ventricular dysfunction	Metabolic regulation	[121]
	Mgat1 (CM)	CM-specific KO	Dilated cardiomyopathy, fibrosis	Metabolism/remodeling	[122]
	Mgat4a	KO	Hyperglycemia, insulin resistance (no structural phenotype)	Systemic metabolism	[123]
	Mgat5	KO	Altered glucagon sensitivity (no structural phenotype)	Metabolic signaling	[124]
	Neuraminidase 1 (Neu1)	KO	Growth impairment, premature mortality	Development/metabolism	[125]
	Neuraminidase 2 (Neu2)	KO	Dyslipidemia (no defined cardiac phenotype)	Systemic metabolism	[126]
	Neuraminidase 3 (Neu3)	KO	No cardiac phenotype	—	[127]
	EOGT	KO	No obvious cardiac phenotype	—	[128]
Proteoglycans and ECM Glycoproteins	Agrin (Agrn)	KO	Embryonic lethality, epicardial disruption	Development	[129]
	Glypican 3 (Gpc3)	KO	Perinatal lethality, congenital cardiac defects	Development	[130]
	Glypican 1 (Gpc1)	KO	Increased cardiac output, prolonged QT interval	Electrical/functional	[131]
	Perlecan (Hspg2)	KO	Embryonic lethality; increased ischemic susceptibility	Development/ischemia	[132]
	Decorin (Dcn)	KO	Severe post-MI remodeling, impaired autophagy	Remodeling	[133]
	Biglycan (Bgn)	KO	Exacerbated remodeling post-MI/pressure overload	Remodeling	[134]
	Lumican (Lum)	KO	Perinatal death, cardiomyocyte hypertrophy	Development/hypertrophy	[135]
	Fibromodulin (Fmod)	KO	Exacerbated hypertrophy under pressure overload	Remodeling	[136]
	Osteoglycin (Ogn)	KO	Altered hypertrophic response, increased mortality	Remodeling	[137]
	Brevican (Bcan)	KO	No cardiac phenotype	—	[138]
	Proteoglycan 4 (Prg4)	KO	No cardiac phenotype	—	[139]
	Bikunin (Ambp/Bik)	KO	No baseline phenotype	—	[140]
Matricellular and Signaling Modulators	CCN1 (Ccn1)	KO	Embryonic lethality due to cardiac defects	Development	[141]
	CCN2 (Ccn2/Ctgf)	KO/TG	Perinatal lethality; modulated hypertrophy	Development/remodeling	[142,143]
	CCN3 (Ccn3/Nov)	KO	Ventricular dilation, developmental defects	Development	[144]
	CCN5 (Ccn5)	TG	Reduced pressure overload-induced remodeling	Remodeling	[145]
	Asporin (Aspr)	KO	Reduced tolerance to ischemia/reperfusion	Ischemia response	[146]
	CD44 (Cd44)	KO	Reduced Ang II-induced remodeling	Remodeling/inflammation	[147]
	Syndecan-4 (Sdc4)	KO	Blunted exercise-induced hypertrophy	Adaptive remodeling	[148]
	Basigin (Bsg)	Het	Attenuated pressure overload remodeling	Remodeling	[149]
Structural/ Membrane Complex Proteins	Sarcoglycan complex (Sgcb, Sgcg, Sgcd)	KO	Cardiomyopathy, fibrosis, necrosis	Structural integrity	[150,151]
	α/ε-Sarcoglycan (Sgca/Sgce)	KO	Cardiomyopathy; no baseline phenotype early	Structural/functional	[152]
Vascular and Angiogenic ECM Components	Collagen XV (Col15a1)	KO	Capillary collapse; adverse remodeling	Vascular integrity	[153]
	Collagen XVIII (Col18a1)	KO	Increased permeability, enhanced atherosclerosis	Vascular function	[154]
	CSPG4/NG2 (Cspg4)	KO	Reduced angiogenesis, impaired microvasculature	Angiogenesis	[155]

CM: cardiomyocytes; KO: knockout; TG: transgenic; CCN1: CCN family member 1; CCN2: CCN family member 2; CCN5: CCN family member 5; Galnt1: Polypeptide N acetyl-galactosaminyl-transferase 1; Mgat1: Mannoside acetyl-glucosaminyl-transferase 1; Mgat5: Mannoside acetyl-glucosaminyl-transferase 5; Mgat4a: Mannoside acetyl-glucosaminyl-transferase 4a; EOGT: EGF domain specific O linked N acetylglucosamine transferase. This table summarizes in vivo models involving glycosylation enzymes, proteoglycans, and extracellular matrix-associated proteins that influence cardiovascular phenotypes. While not all entries directly regulate O-GlcNAcylation, they are included to highlight broader glycosylation-dependent and matrix-associated mechanisms relevant to cardiovascular physiology and disease. This table does not include all transgenic and knockout glycoprotein models; it comprises of those having cardiac phenotypes or studied for cardiac abnormalities along with the those discussed in the main body of the article.

**Table 2:** Emerging technologies for studying O-GlcNAc with relevance to Acute and Chronic remodeling.

Tool Category	Method	Relevance to Acute vs. Chronic Studies
Chemical labeling	Click chemistry	Captures global O-GlcNAc changes at defined time points
Targeting tools	OGT + RNA aptamers	Enables acute, protein-specific modulation of O-GlcNAc
Biosensors	FRET/BRET sensors	Real-time tracking of dynamic (acute) O-GlcNAc changes
Proximity labeling	GlycoID (BirA *)	Identifies temporal interaction networks
Targeted modulation	Nanobody–OGT/OGA	Distinguishes acute vs. sustained modification effects
Optogenetics	Photo-OGT	Precise temporal control (ideal for acute vs. chronic comparison)
Protein engineering	Engineered OGT systems	Models prolonged (chronic) O-GlcNAc elevation

*BirA* denotes an engineered/promiscuous biotin ligase used for proximity-dependent protein labeling. The asterisk (*) indicates a modified mutant form of the native BirA enzyme. In the context of GlycoID, BirA* helps label proteins associated with O-GlcNAc-containing complexes or microenvironments, enabling identification of temporal and spatial interaction networks.

**Table 3:** Emerging Needs and Opportunities in O-GlcNAc Biology.

Category	Focus Area	Key Elements	Goal/Outcome
Data (Discovery)	Build O-GlcNAc atlases	Site mapping; stoichiometry; cross-cell datasets	Comprehensive and quantitative O-GlcNAc profiling
Tool Development	Enable high-throughput discovery	CRISPR screens; phage display; directed evolution	Scalable identification of regulators and substrates
Mechanism	Define molecular mechanisms	OGT/OGA enzymes; reader proteins; structural data	Mechanistic understanding of O-GlcNAc regulation
Targeting	Develop selective modulators	Ligands; OGT/OGA-adjacent targets	Increased specificity beyond global inhibition
Dynamics & Disease	Decode spatiotemporal regulation	Temporal dynamics; pathway feedback; disease links	Context-specific and disease-relevant insights

## Data Availability

No new data were created or analyzed in this study. Data sharing is not applicable to this article.
